# ICTV Virus Taxonomy Profile: *Ovaliviridae*

**DOI:** 10.1099/jgv.0.001546

**Published:** 2020-12-17

**Authors:** Li Huang, Haina Wang, ICTV Report Consortium

**Affiliations:** ^1^​ State Key Laboratory of Microbial Resources, Institute of Microbiology, Chinese Academy of Sciences, Beijing 100101, PR China; ^2^​ College of Life Sciences, University of Chinese Academy of Sciences, Beijing 100049, PR China; ^3^​ School of Water Resources and Environment, China University of Geosciences, Beijing 100083, PR China

**Keywords:** ICTV Report, *Ovaliviridae*, taxonomy

## Abstract

*Ovaliviridae* is a family of enveloped viruses with a linear dsDNA genome. The virions are ellipsoidal, and contain a multi-layered spool-like capsid. The viral genome is presumably replicated through protein priming by a putative DNA polymerase encoded by the virus. Progeny virions are released through hexagonal openings resulting from the rupture of virus-associated pyramids formed on the surface of infected cells. The only known host is a hyperthermophilic archaeon of the genus *Sulfolobus *. This is a summary of the International Committee on Taxonomy of Viruses (ICTV) Report on the family *Ovaliviridae,* which is available at ictv.global/report/ovaliviridae.

## Virion

**Fig. 1.**
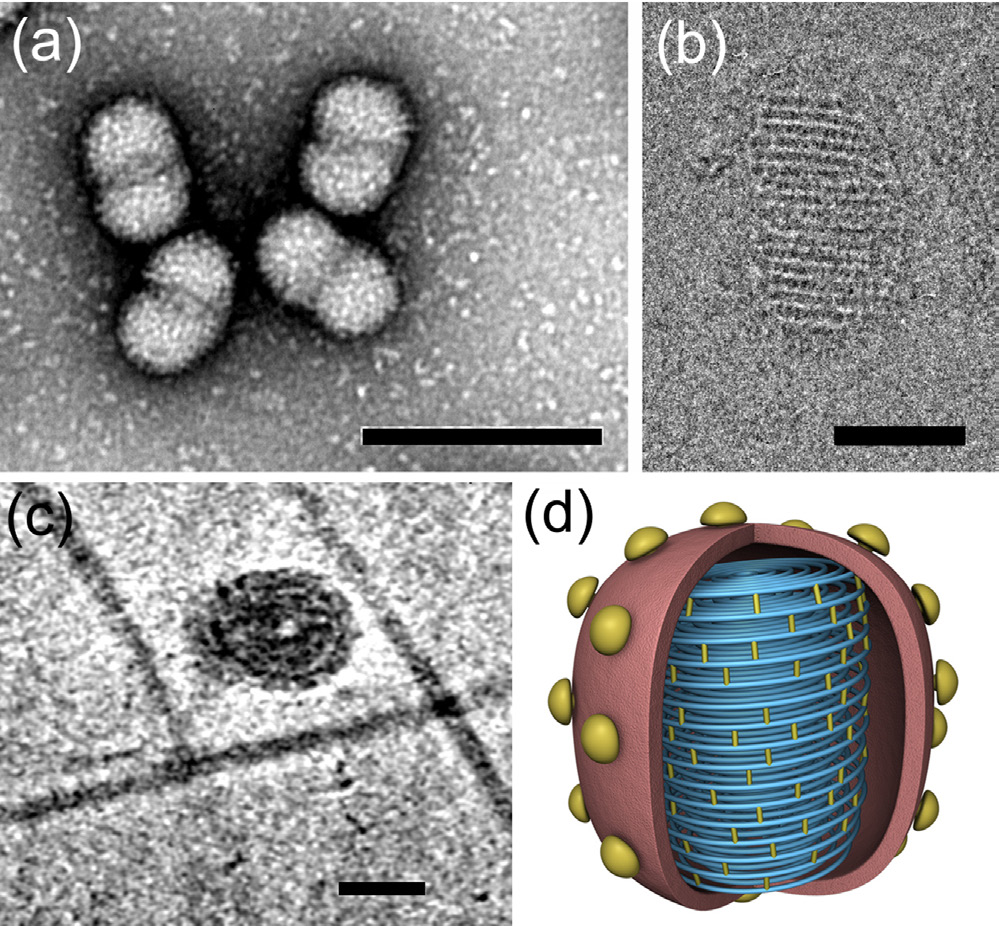
Virions of Sulfolobus ellipsoid virus 1. (a) Negative-stain electron micrograph (bar, 200 nm). (b) Cryo-electron micrograph (bar, 50 nm). (c) Top view (bar, 50 nm). (d) Schematic three-dimensional model (modified from [1]).

Virions of Sulfolobus ellipsoid virus 1 are ellipsoidal, about 115×78 nm and coated with an envelope (Table 1, Fig. 1). Constriction in the middle of the virion is observed under negative-stain transmission electron microscopy, presumably due to dehydration of the virus particle during sample preparation (Fig. 1a). Under cryo-electron microscopy, 16 helical striations aligned perpendicular to the longitudinal axis of the virion with a periodicity of about 5 nm are clearly visible (Fig. 1b). Each electron-dense stripe is about 2.8 nm wide. The longitudinal section of a virion obtained by three-dimensional cryo-electron tomography reveals a tube-like structure of about 8 nm in diameter at the centre of the capsid (Fig. 1c, d). Striations are also evident in the interior of the virion. The capsid is probably formed by coiling of a nucleoprotein ﬁlament. The ﬁlament wraps tightly around the longitudinal axis multiple times in a plane to form a disc-like structure (Fig. 1c). Sixteen of the stacked discs constitute the capsid (Fig. 1d). Isoprenoid glycerol dibiphytanyl glycerol tetraethers in the virion envelope are similar to those in the host cell membrane in composition. Four major structural proteins of 12–170 kDa exist in the virus particles [1].

**Table 1.** Characteristics of members of the family *Ovaliviridae*

Example:	Sulfolobus ellipsoid virus 1 (MF144115), species *Sulfolobus ellipsoid virus 1*, genus *Alphaovalivirus*
Virion	Ellipsoid; 115 nm long, 78 nm wide; an envelope encases a striated capsid
Genome	Linear, dsDNA (23 219 bp) with 172 bp inverted terminal repeats
Replication	Possible protein-primed DNA replication, lytic
Translation	Uncharacterized
Host range	Hyperthermophilic archaea of the genus *Sulfolobus *
Taxonomy	One genus with a single species

## Genome

**Fig. 2.**
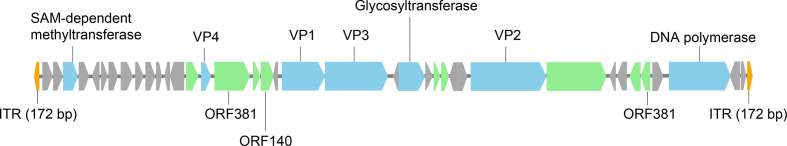
Sulfolobus ellipsoid virus 1 genome organization. The size and direction of each predicted ORF or genetic element are indicated by an arrow. Colours indicate ORFs with a putative function (blue), those homologous to ORFs from other archaeal viruses or containing conserved motifs (green), and those having no significant sequence matches to known sequences in public databases (grey). The inverted terminal repeats (ITRs) are coloured orange [1].

The Sulfolobus ellipsoid virus 1 virion contains a linear dsDNA genome of 23 219 bp with 172 bp inverted terminal repeats. The G+C content of the genome is 33%. The genome harbours 38 putative protein-encoding genes (Fig. 2) [1]. The majority (27/38) of the ORFs reside on one of the strands. Most ORFs encode proteins of unknown function. Four genes (*vp1*, *vp2*, *vp3* and *vp4*) encode structural proteins, as verified by the analysis of virus particles by SDS-PAGE. The genome is also predicted to encode a glycosyltransferase, a B-family DNA polymerase and a SAM-dependent methyltransferase. Four ORFs (the ORF for the putative SAM-dependent methyltransferase, ORF381, ORF100 and ORF140) are homologous to sequences in lipothrixviruses or rudiviruses, and the first three of them also to sequences in the spindle-shaped virus Sulfolobus monocaudavirus 1 (an unclassified virus related to members of the family *Bicaudaviridae*), implying possible horizontal gene transfer between these viruses.

## Replication

Sulfolobus ellipsoid virus 1 encodes a putative B-type DNA polymerase belonging to the subfamily of protein-primed DNA polymerases [2]. This polymerase is similar to those from some plasmids and viruses including Acidianus bottle shaped virus (*Ampullaviridae*) [3], His1 virus (*Halspiviridae*) and His2 virus (*Pleolipoviridae*) [4], and Nitrosopumilus spindle-shaped virus 1 (*Thaspiviridae*) [5]. However, no homologue to terminal protein, which is required to prime replication [2], has been identified in Sulfolobus ellipsoid virus 1. Sulfolobus ellipsoid virus 1 infection only slightly retards the growth of the host cells. Progeny virions are released through hexagonal openings resulting from the rupture of virus-associated pyramids formed on the host cell surface.

## Taxonomy

Current taxonomy: ictv.global/taxonomy. Sulfolobus ellipsoid virus 1 was isolated from an acidic hot spring (86–106 °C, pH 2.2–2.5) in Laguna Fumarolica, Costa Rica. The only known host is *Sulfolobus* sp. A20, which was isolated from the same hot spring [6]. The family *Ovaliviridae* includes the genus *Alphaovalivirus* and the species *Sulfolobus ellipsoid virus 1*.

## Resources

Full ICTV Report on the family *Ovaliviridae*: ictv.global/report/ovaliviridae.
